# Radiolabeled F(ab′)_2_-cetuximab for theranostic purposes in colorectal and skin tumor-bearing mice models

**DOI:** 10.1007/s12094-018-1886-4

**Published:** 2018-05-17

**Authors:** P.-S. Bellaye, M. Moreau, O. Raguin, A. Oudot, C. Bernhard, J.-M. Vrigneaud, L. Dumont, D. Vandroux, F. Denat, A. Cochet, F. Brunotte, B. Collin

**Affiliations:** 1 Service de médecine nucléaire, Centre Georges-François Leclerc, 1 rue du professeur Marion, 21000 Dijon, France; 2 Institut de Chimie Moléculaire de l’Université de Bourgogne, UMR CNRS 6302, Université de Bourgogne Franche-Comté, 21078 Dijon Cedex, France; 3 Oncodesign, 21076 Dijon Cedex, France; 4 NVH Medicinal, 64 rue Sully, 21000 Dijon, France

**Keywords:** Cetuximab fragments, Colorectal cancer, Radioimmunotherapy, EGFR, HSP90

## Abstract

**Purpose:**

This study aimed to investigate theranostic strategies in colorectal and skin cancer based on fragments of cetuximab, an anti-EGFR mAb, labeled with radionuclide with imaging and therapeutic properties, ^111^In and ^177^Lu, respectively.

**Methods:**

We designed F(ab′)_2_-fragments of cetuximab radiolabeled with ^111^In and ^177^Lu. ^111^In-F(ab′)_2_-cetuximab tumor targeting and biodistribution were evaluated by SPECT in BalbC nude mice bearing primary colorectal tumors. The efficacy of ^111^In-F(ab′)_2_-cetuximab to assess therapy efficacy was performed on BalbC nude mice bearing colorectal tumors receiving 17-DMAG, an HSP90 inhibitor. Therapeutic efficacy of the radioimmunotherapy based on ^177^Lu-F(ab′)_2_-cetuximab was evaluated in SWISS nude mice bearing A431 tumors.

**Results:**

Radiolabeling procedure did not change F(ab′)_2_-cetuximab and cetuximab immunoreactivity nor affinity for HER1 in vitro. ^111^In-DOTAGA-F(ab′)_2_-cetuximab exhibited a peak tumor uptake at 24 h post-injection and showed a high tumor specificity determined by a significant decrease in tumor uptake after the addition of an excess of unlabeled-DOTAGA-F(ab′)_2_-cetuximab. SPECT imaging of ^111^In-DOTAGA-F(ab′)_2_-cetuximab allowed an accurate evaluation of tumor growth and successfully predicted the decrease in tumor growth induced by 17-DMAG. Finally, ^177^Lu-DOTAGA-F(ab′)_2_-cetuximab radioimmunotherapy showed a significant reduction of tumor growth at 4 and 8 MBq doses.

**Conclusions:**

^111^In-DOTAGA-F(ab′)_2_-cetuximab is a reliable and stable tool for specific in vivo tumor targeting and is suitable for therapy efficacy assessment. ^177^Lu-DOTAGA-F(ab′)_2_-cetuximab is an interesting theranostic tool allowing therapy and imaging.

**Electronic supplementary material:**

The online version of this article (10.1007/s12094-018-1886-4) contains supplementary material, which is available to authorized users.

## Introduction

The epidermal growth factor receptor (EGFR) has evolved over the years into a main molecular target for the treatment of different cancer entities. EGFR is a glycosylated transmembrane protein involved in regulating cell growth, differentiation and survival of malignant cells [1]. This receptor is often overexpressed in various malignancies such as head and neck squamous cell carcinoma (HNSCC), gastrointestinal/abdominal carcinoma, lung and reproductive tract carcinomas, melanomas, glioblastomas and thyroid carcinoma [2]. Despite some controversies, the overexpression of EGFR is often associated with a poor clinical prognosis and resistance to radiation therapy [3, 4]. Therefore, therapeutic strategies involving monoclonal antibodies against EGFR, such as cetuximab, have been used pre-clinically and clinically. Cetuximab is a chimeric monoclonal antibody, which binds specifically with a high affinity to the extracellular domain of the EGFR. Cetuximab is used alone or in combination and its therapeutic indications are (i) colorectal and HNSCC and (ii) HNSCC with external radiotherapy [5]. While the combination of cetuximab with radiotherapy showed improved survival [5], tumor response remains heterogeneous and cetuximab failed to show benefit over chemoradiotherapy [6, 7]. Hence, radioimmunotherapy based on cetuximab labeled with therapeutic radionuclides appears as a promising strategy allowing the delivery of radiation dose specifically to tumor cells expressing high level of EGFR while sparing normal tissues. In this regard, theranostic strategy based on monoclonal antibodies and their deriving structures could represent an exciting approach. This strategy relies on the combination of detection and treatment in one integrated approach [8]. Indeed, it is possible to convert a purely imaging probe into a single-entity theranostic agent by simply switching the radionuclide from a γ-emitter (e.g.: ^111^In) to a β-emitter (e.g.: ^90^Y) or using a radionuclide gathering both imaging and therapeutic properties (e.g.: ^177^Lu). We assume that such agents should be safer, more efficient and innovative through their capabilities in sequentially or simultaneously diagnosing, monitoring, and treating disease. The other major theranostic approach relies on the use of a molecular probe to detect and follow-up a given biomarker during a pharmacotherapy, which is, for example, commonly performed in clinical routine with ^18^F-FDG and PET in oncology [8]. The relevance of using cetuximab radiolabeled with ^111^In in diagnostic imaging has been demonstrated in several animal models mainly due to its high tumor targeting property and its adequate half-life [9–12]. ^177^Lu is a theranostic radionuclide of choice to target small tumors or metastatic deposits due to the medium energy of the emitted beta particles and their tissue penetration of 1.5 mm. In addition, ^177^Lu emits γ-rays which allow diagnostic imaging. The therapeutic efficacy of cetuximab radiolabeled with ^177^Lu has been demonstrated on various tumor types [13–16].

A major concern with the use of a β-emitter such as ^177^Lu as radionuclide is the selection of a chelating agent that forms a sufficiently stable complex to prevent in vivo loss of the radiometal. This choice is crucial to avoid toxicity induced by the delivery of undesired amount of radiation mainly to the radio sensitive bone marrow. Several bifunctional chelating agents (BFCA) have been used for the radiolabeling of cetuximab including DTPA and DOTA derivatives. Several reports highlight the higher in vitro and in vivo stability of DOTA conjugates to sequester ^111^In, ^177^Lu but also ^90^Y, resulting in lower bone incorporation and marrow toxicity [14, 17–21]. Our lab previously made the proof of concept that trastuzumab and cetuximab radiolabeled with ^111^In through our new bifunctional chelating agent DOTAGA anhydride [22], were able to bind to their target both in vitro and in vivo [23]. Nevertheless, full-sized antibodies (about 150 kDa) need to overcome some obstacles before penetrating into a tumor. Indeed, tumor penetration can be hampered by some physiological barriers, such as high interstitial pressure and a “binding site barrier” [24, 25]. To circumvent these drawbacks, it is possible to generate fragments [Fab and F(ab′)_2_] of the monoclonal antibody to improve its penetration within the tumor. These fragments can be prepared through enzymatic cleavages [papain for Fab and pepsin for F(ab′)_2_] or by genetic engineering. Given their pharmacokinetic properties, F(ab′)_2_ (110 kDa) could be valuable theranostic agents. Indeed, they are bivalent as the native monoclonal antibody but they are less immunogenic, show a shorter blood clearance, higher tumor-to-background ratios and reduced non-specific distribution [26].

In the present work, our aims were to design F(ab′)_2_-cetuximab-based theranostic agents with both diagnostic and therapeutic capabilities and to assess them in murine preclinical cancer models. First, we characterized in vitro the F(ab′)_2_ fragment of cetuximab radiolabeled with ^111^Indium (^111^In-DOTAGA-F(ab′)_2_-cetuximab) in comparison with whole cetuximab used as a reference (^111^In-DOTAGA-cetuximab). Then, we evaluated the stability of DTPA- and DOTAGA-radiolabeled F(ab′)_2_-cetuximab. We made the proof of concept that ^111^In-DOTAGA-F(ab′)_2_-cetuximab was suitable for monitoring the down-regulation of EGFR as a biomarker of the efficacy of a targeted therapy with a HSP90 inhibitor. In cancer cells, the stability of EGFR is promoted by the chaperon protein HSP90 [27]. The chemical inhibition of HSP90 leads to an increase in EGFR degradation hampering oncologic signaling pathways [27]. Finally, we designed a strategy based on radioisotopic switching to perform radioimmunoscintigraphy (^111^In-DOTAGA-F(ab′)_2_-cetuximab) prior to radioimmunotherapy (^177^Lu-DOTAGA-F(ab′)_2_-cetuximab) in colorectal tumor-bearing mice. ^177^Lu-DOTAGA-F(ab′)_2_-cetuximab showed a good tolerance and efficacy for reducing tumor volume in our model.

## Materials and methods

Detailed Materials and methods are available in Supplemental materials.

### Preparation of cetuximab and F(ab′)_2_ fragments

F(ab′)_2_ fragment was prepared by dialysis of 15 mL of cetuximab at 5 mg.mL^− 1^ against pepsin buffer (sodium acetate 20 mM/acetic acid pH4) on 10 kDa cut off (Amicon Ultra 15—Millipore) using centrifugation (4000 *g*; 2 × 20 min). See details in Supplemental methods.

### Derivatization of cetuximab and F(ab′)_2_ fragments for radiolabeling

#### Conjugation of DOTAGA anhydride with cetuximab

Conjugation was performed at a 20:1 DOTAGA-anhydride/cetuximab molar ratio. 100 μL of a 3.7 mg.mL^− 1^ suspension of DOTAGA-anhydride (370 μg, 0.8 μmol, 20 equiv) in dry chloroform (Carlo Erba, Val de Reuil, France) were pipetted under ultrasonication and transferred into a 15 mL polypropylene tube. The chloroform was evaporated under a gentle stream of air. 480 μL of a solution of purified cetuximab (12.5 mg.mL^− 1^, 6 mg, 40 nmol, 1 equiv) in PBS, pH 7.4, (Fisher Scientific, Illkirch, France) were subsequently added. The solution was completed to 3 mL with PBS 0.1 M, pH 7.4, and gently mixed at 25 °C for 30 min. Unbound DOTAGA was then removed by ultrafiltration (Vivaspin filter 10 kDa, Sartorius, 30 min at 1520 g, 4 °C). Conjugated cetuximab was washed twice with 5 mL of PBS 0.1 M, pH 7.4, and the concentrated solution was diluted in 500 μL of ammonium acetate buffer 0.1 M pH 5.9. The purified immunoconjugate DOTAGA-cetuximab was stored at 4 °C. Concentration of the antibody was determined by UV spectrophotometry at 280 nm (*E*_280_ = 1.44 mg mL^− 1^ cm^− 1^). The degree of labeling was determined by MALDI-TOF mass spectrometry using sinapinic acid as matrix.

#### Conjugation of DOTAGA anhydride to F(ab′)_2_-cetuximab

Following a similar procedure, conjugation was performed at a 15:1 DOTAGA-anhydride/F(ab′)_2_ molar ratio. 111 μL of a 3.7 mg.mL^− 1^ suspension of DOTAGA-anhydride (411 μg, 0.9 μmol, 15 equiv) in dry chloroform were pipetted under ultrasonication and transferred into a 15 mL polypropylene tube. See details in Supplemental methods.

### ^111^In and ^177^Lu radiolabeling procedures

#### General procedure for radiolabeling for in vitro studies

7.5 MBq of ^111^InCl_3_ (Perkin Elmer) were added to 50 μg of the immunoconjugate in 0.1 M ammonium acetate buffer, pH 5.9, to reach a buffer/HCl (from ^111^InCl_3_ solution) ratio of 1.5:1 resulting in a pH 5 solution. See details in Supplemental methods.

#### General procedure for radiolabeling for in vivo studies

^111^InCl_3_ or ^177^LuCl_3_ (Perkin Elmer) were buffered with 1/10th (v/v) of 1 M ammonium acetate solution pH 7.1 and then added to DOTAGA-F(ab′)_2_ in 0.1 M ammonium acetate buffer pH 5.9 (600 MBq mg^− 1^ and 1 GBq.mg^− 1^, respectively). See details in Supplemental methods.

### Stability assay

^111^In-DOTAGA-F(ab′)_2_-cetuximab/^111^In-DTPA-F(ab′)_2_-cetuximab stability in EDTA and plasma were evaluated up to 7 days. See details in Supplemental methods.

### Cell culture

Epidermoid carcinoma cells A431 (ATCC, Rockville, MD) overexpressing the HER1 antigen at their surface [28] and primary human tumor fragments from colon tumor (CR-LRB-014P) have been used. CR-LRB-014P cells have been collected from a biopsy of a primitive colorectal tumor from a 70-year-old male in 2008 in the hospital center of “Lariboisière”, Paris, France (no treatment before surgery). See details in Supplemental methods.

### Determination of ^111^In-DOTAGA-cetuximab and ^111^In-DOTAGA-F(ab′)_2_-cetuximab binding affinity and immunoreactivity

#### Immunoreactivity

The fraction of ^111^In-DOTAGA-F(ab′)_2_-cetuximab and ^111^In-DOTAGA-cetuximab able to bind to HER1 was determined by incubating trace amounts of ^111^In-DOTAGA-F(ab′)_2_-cetuximab or ^111^In-DOTAGA-cetuximab (1 MBq, 7.0 × 10^− 9^ M) with increasing concentrations of HER1 expressing cell A431 (0.4–24 × 10^6^ cells mL^− 1^) in a total volume of 0.2 mL for 1 h at 4 °C. See details in Supplemental methods.

#### Binding affinity

The affinity constants (Kd) of the ^111^In-DOTAGA-F(ab′)_2_-cetuximab and ^111^In-DOTAGA-cetuximab were determined in radioligand binding saturation assays. Approximately 3 × 10^5^ A431 cells were incubated with increasing concentrations of ^111^In-DOTAGA-F(ab′)_2_-cetuximab or ^111^In-DOTAGA-cetuximab (5.3 × 10^− 11^ to 5.4 × 10^− 8^ M, 100 MBq·mg^− 1^) in a total volume of 0.2 mL for 1 h at 4 °C. See details in Supplemental methods.

### Animal models

All animal experiments were performed according to the guidelines of the Ministère de la Recherche (Paris, France). All experiments were approved by the ethical committee of the “Centre Georges-François Leclerc” (Dijon, France).

### Biodistribution study

#### SPECT/CT imaging protocol and γ-counting

Female Balb/c nu/nu mice (*n* = 3, 6–8 weeks old, purchased from Charles River, France) were grafted by subcutaneous injection of colon tumor fragments from human patients (CR-LRB-014P). When grown these tumors were collected and fragments from these tumors were implanted into a second set of Balb/c nu/nu mice (6–8 weeks old). 3 or 5 weeks after tumor implantation, tumor-bearing mice were given 25 μg ^111^In-DOTAGA-F(ab′)_2_-cetuximab (13–15 MBq) by intravenous injection. In a second experiment to assess the specificity of the targeting in vivo, a group of mice received 25 μg ^111^In-DOTAGA-F(ab′)_2_-cetuximab (3–3.5 MBq) in co-injection with excess (2500 μg) cold-F(ab′)_2_-cetuximab. Two groups of mice were then studied: (1) ^111^In-DOTAGA-F(ab′)_2_-cetuximab and (2) ^111^In-DOTAGA-F(ab′)_2_-cetuximab + non radiolabelled-DOTAGA-F(ab′)_2_-cetuximab. SPECT/CT dual imaging was performed 3, 6, 20, 24, 48, and 72 h after the injection of the radiolabeled conjugate using a NanoSPECT/CT small animal imaging tomographic γ-camera (Bioscan Inc., Washington, DC). Mice were anaesthetized with isoflurane (1.5–3% in air) and positioned in a dedicated cradle. CT and SPECT acquisitions were performed in immediate sequence. CT acquisitions (55 kVp, 34 mAs) were first acquired during 15–20 min, followed by helical SPECT acquisitions with 90–120 s per projection frame resulting in acquisition times of 45–60 min. Both indium-111 photopeaks (171 and 245 keV) were used with 10% wide energy windows. After the last image acquisition, animals were euthanized. Blood, tumor, and organs were collected, and radioactivity was measured with a scintillation γ-counter. Data were then converted to percentage of injected dose and to percentage of injected dose per mm^3^ of tissue. The CT and SPECT reconstructions were performed using image processing software provided by Bioscan Inc. Eventually, the SPECT/CT fusion image was obtained using the InVivoScope software (Bioscan Inc.). Each scan was then visually interpreted, and 3D regions of interest corresponding to the tumor and whole body were manually drawn to determine their radioactivity content. In vivo quantification was obtained by accurate calibration of the NanoSPECT/CT γ-camera. Radioactivity contents from image analysis were expressed in Bq/mm^3^, converted to percentage of injected dose, and compared to those determined by ex vivo counting.

### Therapy evaluation study

#### ^111^In-DOTAGA-F(ab′)_2_-cetuximab to evaluate HSP90 inhibition

Female Balb/c nu/nu mice (*n* = 10, 6–8 weeks old) were grafted by subcutaneous injection of colon tumor fragments from human patients. When grown these tumors were collected and fragments from these tumors were implanted into a second set of Balb/c nu/nu mice (6–8 weeks old). Tumor volume was measured three times a week from D30 to D50 after cells injection. Mice were randomized in two groups with (1) mice receiving i.p. 17-DMAG (17-DMAG group, 25 mg/kg) or (2) vehicle (vehicle group) three times a week from D30. ^111^In-F(ab′)_2_-cetuximab tumor uptake (iv. injection) was evaluated every week from D30 using SPECT-CT imaging (20–25 μg, 10 MBq, imaging 24 h after injection) as described above (D38, D44, D51 and D58).

### Targeted radioimmunotherapy study

#### Dose escalation of ^177^Lu-DOTAGA-F(ab′)_2_-cetuximab in vivo

Under isoflurane anesthesia, female SWISS nu/nu mice (*n* = 4) were grafted by subcutaneous injection in the flank with 2 × 10^7^ A431 cells. Tumor volume was measured three times a week from D3 after administration. At D14 after tumor cell injection, mice were randomized into four groups with (1) mice receiving vehicle (vehicle group), (2) mice receiving ^177^Lu-DOTAGA-F(ab′)_2_-cetuximab at 2 MBq (2 MBq group), (3) mice receiving ^177^Lu-DOTAGA-F(ab′)_2_-cetuximab at 4 MBq (4 MBq group) and (4) mice receiving ^177^Lu-DOTAGA-F(ab′)_2_-cetuximab at 8 MBq (8 MBq group). ^177^Lu-DOTAGA-F(ab′)_2_-cetuximab was injected i.v. (vehicle, 2 MBq, 4 MBq or 8 MBq/mouse). SPECT-CT imaging was performed 24 h after i.v. injection as described above to assess tumor targeting. Tumor volume was then assessed three times a week up to D23. Weight loss of animals was monitored throughout the experiments.

### Statistical analysis

All results are presented as mean ± SEM. A *p* value less than 0.05 was considered significant. See details in Supplemental methods.

## Results

### DOTAGA-cetuximab and DOTAGA-F(ab′)_2_-cetuximab retain their immunoreactivity and affinity for HER1

**Fig. 1:**
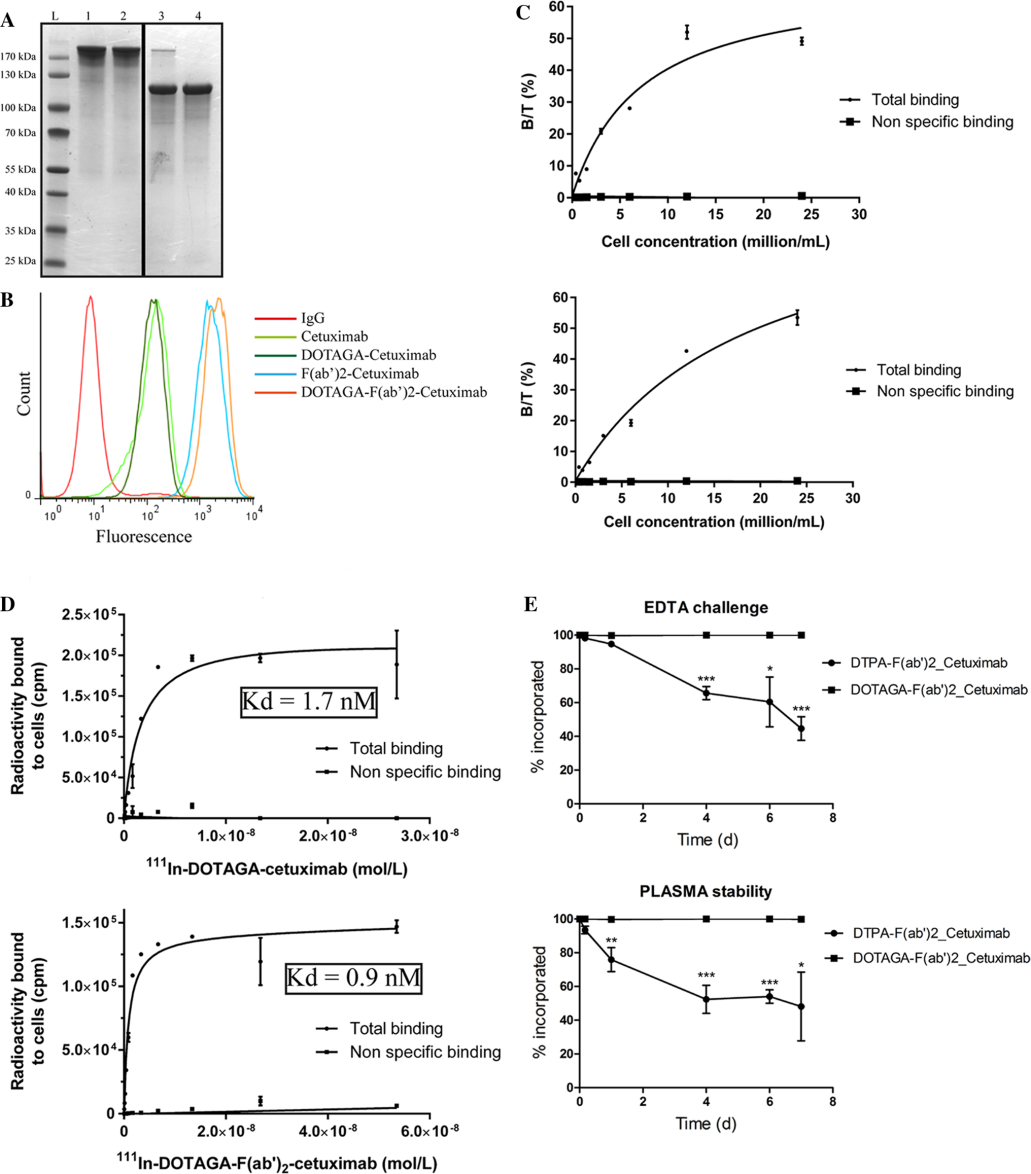
DOTAGA-cetuximab and DOTAGA-F(ab′)_2_-cetuximab retain their immunoreactivity and affinity for HER1. **a** 4–12% bis–tris acrylamide gel stained with coomassie blue performed on 5 µg of whole cetuximab (1), whole cetuximab after dialysis (2), F(ab′)_2_ fragments after digestion (8 h at 37 °C) (3) and F(ab′)_2_ fragments after purification on protein A and *L* columns and dialysis (4). *L* = Protein Ladder. **b** FACS analysis of A431 fluorescence incubated with cetuximab (light green), DOTAGA-cetuximab (dark green), F(ab′)_2_-cetuximab (light blue), DOTAGA-F(ab′)_2_-cetuximab (orange). Non-relevant IgG served as control. **c** Immunoreactivity assay of ^111^In-DOTAGA-cetuximab (higher panel) and ^111^In-DOTAGA-F(ab′)_2_-cetuximab (lower panel). 1 MBq of ^111^In-DOTAGA-cetuximab or ^111^In-DOTAGA-F(ab′)_2_-cetuximab were incubated with increasing concentration of A431 cells (0.4–24 × 10^6^). The radioactivity associated to cells (bound radioactivity, B) and an aliquot of the supernatant (total radioactivity, T) to calculate the bound-to-total ratios (B/T, expressed in %). Nonspecific binding was evaluated in the presence of *a* > 100-fold excess unlabeled cetuximab or F(ab′)_2_-cetuximab. Immunoreactivity was defined as the highest B/T% ratio that could be reached. Results are presented as mean ± SEM, *n* = 3. **d** Binding affinity assay of ^111^In-DOTAGA-cetuximab (higher panel) and ^111^In-DOTAGA-F(ab′)_2_-cetuximab (lower panel). 3 × 10^5^ A431 cells were incubated with increasing concentrations of ^111^In-DOTAGA-F(ab′)_2_-cetuximab or ^111^In-DOTAGA-cetuximab (5.3 × 10^− 11^–5.4 × 10^− 8^ M, 100 MBq·mg^− 1^). Radioactivity associated to cells was counted with a scintillation γ-counter. Nonspecific binding was evaluated in the presence of *a* > 100-fold excess unlabeled DOTAGA-F(ab′)_2_-cetuximab or DOTAGA-cetuximab. Results are presented as mean ± SEM, *n* = 3. **e**. EDTA (upper panel) and plasma (lower panel) stability of ^111^In-DOTAGA-F(ab′)_2_-cetuximab and ^111^In-DTPA-F(ab′)_2_-cetuximab from D0 up to D7. Graphs represent % of ^111^In still incorporated in each bioconjugate. Results are presented as mean ± SEM, *n* = 3

We first evaluated the production and purification of F(ab′)_2_-cetuximab by western blotting (Fig. 1a). As expected, dialysis did not disrupt the integrity of cetuximab and dialyzed and non-dialyzed cetuximab whole antibodies presented a similar profile with a molecular weight above 170 kDa. Pepsin digestion of cetuximab was almost complete with a large band corresponding to the size of F(ab′)_2_ fragment near 110–120 kDa and only a light band remaining at 170 kDa. After purification on the two columns and dialysis (yield: 40%), the residual whole antibody was fully eliminated with a purity of F(ab′)_2_-cetuximab greater than 95% (Fig. 1a). Once purified, cetuximab and F(ab′)_2_-cetuximab were placed with a 20- or 15-fold excess of DOTAGA-anhydride for 30 min at 25 °C resulting in conjugation of 3.7 and 3.1 DOTAGA chelators per molecule, respectively. The labeling efficiencies measured by ITLC for ^111^In-DOTAGA-cetuximab, ^111^In-DOTAGA-F(ab′)_2_-cetuximab, and ^177^Lu-DOTAGA-F(ab′)_2_-cetuximab were above 98% (data not shown). The ability of both forms of cetuximab to bind to HER1 was then evaluated by FACS on A431 cells which express this receptor (Fig. 1b). Interestingly, a shift in cell-associated fluorescence was observed by FACS with DOTAGA–cetuximab and DOTAGA–F(ab′)_2_-cetuximab comparable with cetuximab and F(ab′)_2_-cetuximab alone, respectively (Fig. 1b). Thus, the binding of DOTAGA on both forms of cetuximab did not disturb its binding ability on HER1. To confirm these results, the immunoreactivity and affinity of DOTAGA–cetuximab and DOTAGA–F(ab′)_2_-cetuximab have been evaluated on A431 cells. ^111^In-DOTAGA–cetuximab and ^111^In-DOTAGA–F(ab′)_2_-cetuximab have similar immunoreactivity around 50% (Fig. 1c). Moreover, the affinity of ^111^In-DOTAGA–cetuximab was evaluated at 1.7 nM and the affinity of ^111^In-DOTAGA–F(ab′)_2_-cetuximab was 0.9 nM (Fig. 1d). These affinities values were compatible with in vivo use of radioimmunoconjugates. Finally, DOTAGA–F(ab′)_2_-cetuximab was used for our in vivo experiments. All together these results demonstrate that F(ab′)_2_ fragments of cetuximab retain their immunoreactivity and affinity for HER1 which are not disturbed by DOTAGA incorporation.

#### DOTAGA conjugates show much higher stability than DTPA conjugates

^111^In-DOTAGA-F(ab′)_2_-cetuximab and ^111^In-DTPA-F(ab′)_2_-cetuximab stability toward EDTA and human plasma was evaluated on 7 days. The stability of ^111^In-DOTAGA-F(ab′)_2_-cetuximab was greater than ^111^In-DTPA-F(ab′)_2_-cetuximab in both conditions with a 100% of ^111^In still incorporated up to D7. ^111^In-DTPA-F(ab′)_2_-cetuximab stability was significantly lower with only 45 and 48% of ^111^In still incorporated at D7 in EDTA and plasma, respectively (Fig. 1d).

#### In vivo tumor specificity of ^111^In- DOTAGA-F(ab′)_2_-cetuximab

**Fig. 2:**
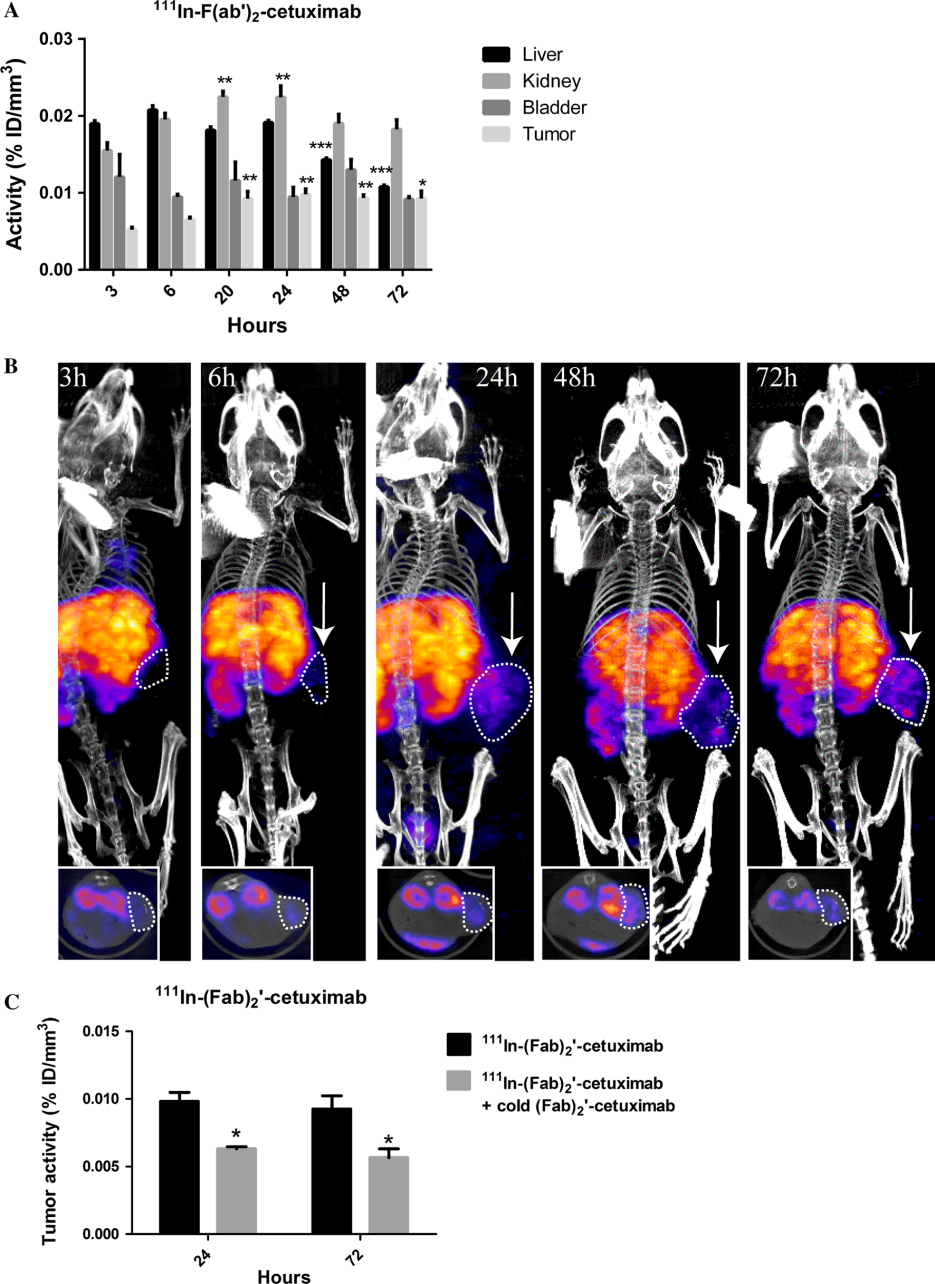
In vivo tumor specificity of ^111^In- DOTAGA-F(ab′)_2_-cetuximab. **a** In vivo biodistribution of ^111^In-DOTAGA-F(ab′)_2_-cetuximab in tumor (CR-LRB-014P cells) bearing balb/c nude mice at 3, 6, 20, 24, 48 and 72 h post injection. Liver, kidneys, bladder and tumor activity are expressed as % ID/mm^3^. Results are presented as mean ± SEM; *n* = 3, **p < 0.01, ***p < 0.001. **b** Representative SPECT pictures of ^111^In-DOTAGA-F(ab′)_2_-cetuximab in tumor (CR-LRB-014P cells) bearing balb/c nude mice at 3, 6, 24, 48 and 72 h post injection. White arrows/circle = tumors. **c** Specific ^111^In-DOTAGA-F(ab′)_2_-cetuximab tumor activity (% ID/mm^3^) of tumor (CR-LRB-014P cells) bearing balb/c nude mice at 24 and 72 h post injection. Co-injection of ^111^In-DOTAGA-F(ab′)_2_-cetuximab with excess (2500 μg) cold-F(ab′)_2_-cetuximab was used to assess specificity. Results are presented as mean ± SEM; *n* = 3, *p < 0.05

^111^In-DOTAGA-F(ab′)_2_-cetuximab biodistribution was evaluated in Balb/c nude mice grafted subcutaneously with CR-LRB-014P human colon tumor fragments at 3, 6, 20, 24, 48 and 72 h post injection. Liver and kidney were the normal tissues with highest uptake (Fig. 2a). The liver uptake remained stable from 3 to 24 h post injection. Then, it started to significantly decrease at 48 and 72 h post injection. The kidney uptake significantly increased at 20 and 24 h compared to 3 h post injection and remained stable up to 72 h post injection. The bladder uptake remained stable throughout the experiment (Fig. 2a). Interestingly, the tumor uptake started to significantly increase at 20 h post injection and remained increased up to 72 h compared to 3 h post injection (Fig. 2a, b). Mice that were pre-injected with an excess of unlabeled F(ab′)_2_-cetuximab had significantly lower tumor uptake demonstrating the specificity of F(ab′)_2_-cetuximab for the CR-LRB-014P tumors (Fig. 2c).

## ^111^In-DOTAGA-F(ab′)_2_-cetuximab is a reliable tool to monitor 17-DMAG treatment efficacy

**Fig. 3:**
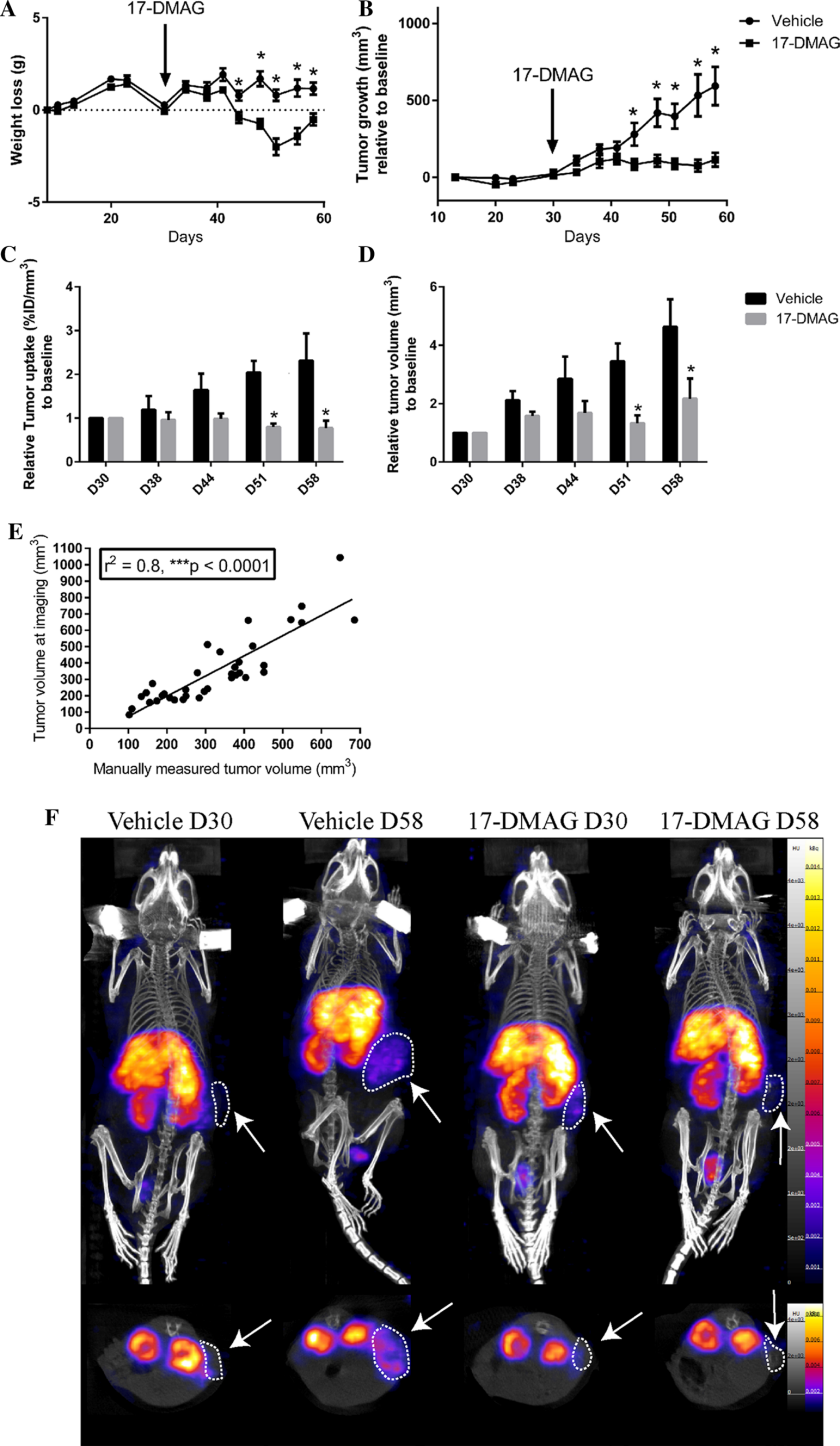
^111^In-DOTAGA-F(ab′)_2_-cetuximab is a reliable tool to monitor 17-DMAG treatment efficacy. **a** Weight loss of Balb/c nude mice receiving vehicle or 17-DMAG. CR-LRB-014P tumors were subcutaneously implanted at D0 and vehicle or 17-DMAG injections were performed starting at D30 (three times a week ip, 25 mg/kg). Results are presented as mean ± SEM; *n* = 10, *p < 0.05. **b** Tumor volume of Balb/c nude mice receiving vehicle or 17-DMAG. CR-LRB-014P tumors were subcutaneously implanted at D0 and vehicle or 17-DMAG injections were performed starting at D30 (three times a week ip, 25 mg/kg). Tumor growth is expressed as a relative gain of volume from the first day when tumor was measurable (D10). Results are presented as mean ± SEM; *n* = 10, *p < 0.05. **c** Relative ^111^In-DOTAGA-F(ab′)_2_-cetuximab (imaging 24 h pi) tumor uptake (% ID/mm^3^) of Balb/c nude mice receiving vehicle or 17-DMAG. Tumor uptake is expressed relative to measures at D30 (start of 17-DMAG treatment). Results are presented as mean ± SEM; *n* = 4, *p < 0.05. **d** Relative tumor volume (mm^3^) measured by SPECT of Balb/c nude mice receiving vehicle or 17-DMAG. Tumor volume is expressed relative to measures at D30 (start of 17-DMAG treatment). Results are presented as mean ± SEM; *n* = 4, *p < 0.05. **e** Correlation between tumor volumes measured manually and measured by SPECT/CT in Balb/c nude mice receiving vehicle or 17-DMAG. **f** Representative SPECT/CT pictures of ^111^In-DOTAGA-F(ab′)_2_-cetuximab in tumor bearing balb/c mice receiving vehicle or 17-DMAG at D30 (at treatment start = baseline) and D58 (4 weeks of treatment). White arrows/circles highlight s.c. tumors

Balb/c nude mice grafted subcutaneously with human primary colon tumor fragments (CR-LRB-014P) received 17-DMAG three times a week from D30 up to D58. Weight loss and tumor growth were monitored throughout the experiment. Mice received ^111^In-F(ab′)_2_-cetuximab every week from D30 (D38, D44, D51 and D58) for SPECT-CT imaging (imaging 24 h after injection). 17-DMAG induced a slight weight loss from D42 (12 days after treatment start) that was partially recovered by D58 compared to mice receiving vehicle (Fig. 3a). Interestingly, 17-DMAG induced a significant decrease in tumor growth compared to vehicle from D44 up to D58 (Fig. 3b). In parallel, tumor uptake of ^111^In-F(ab′)_2_-cetuximab measured by SPECT-CT imaging demonstrated that 17-DMAG treatment reduced tumor uptake compared to vehicle. Although only significant from D58, a marked reduced tumor uptake was observed from D44 (*p* = 0.059, Fig. 3c). Interestingly, tumor volume measured by SPECT-CT imaging reflected the decrease in tumor growth induced by 17-DMAG observed by manual measurements (Fig. 3d–f). However, a significant difference in tumor volume measured by SPECT-CT between 17-DMAG and vehicle was only observed at D51 while already observed at D44 by manual measurements (Fig. 3d). However, the decrease in tumor volume measured by SPECT-CT was clearly observed from D44 even though not significant (*p* = 0.065). Interestingly, there was a strong positive correlation between tumor volumes measured manually and with SPECT/CT imaging (Fig. 3e). All together these results confirm that 17-DMAG prevents tumor growth in our model of CR-LRB-014P tumors. The effectiveness of 17-DMAG can be accurately measured by SPECT-CT imaging of ^111^In-F(ab′)_2_-cetuximab tumor uptake and corresponding tumor volume.

## ^177^Lu-DOTAGA-F(ab′)_2_-cetuximab prevents tumor growth in vivo

**Fig. 4:**
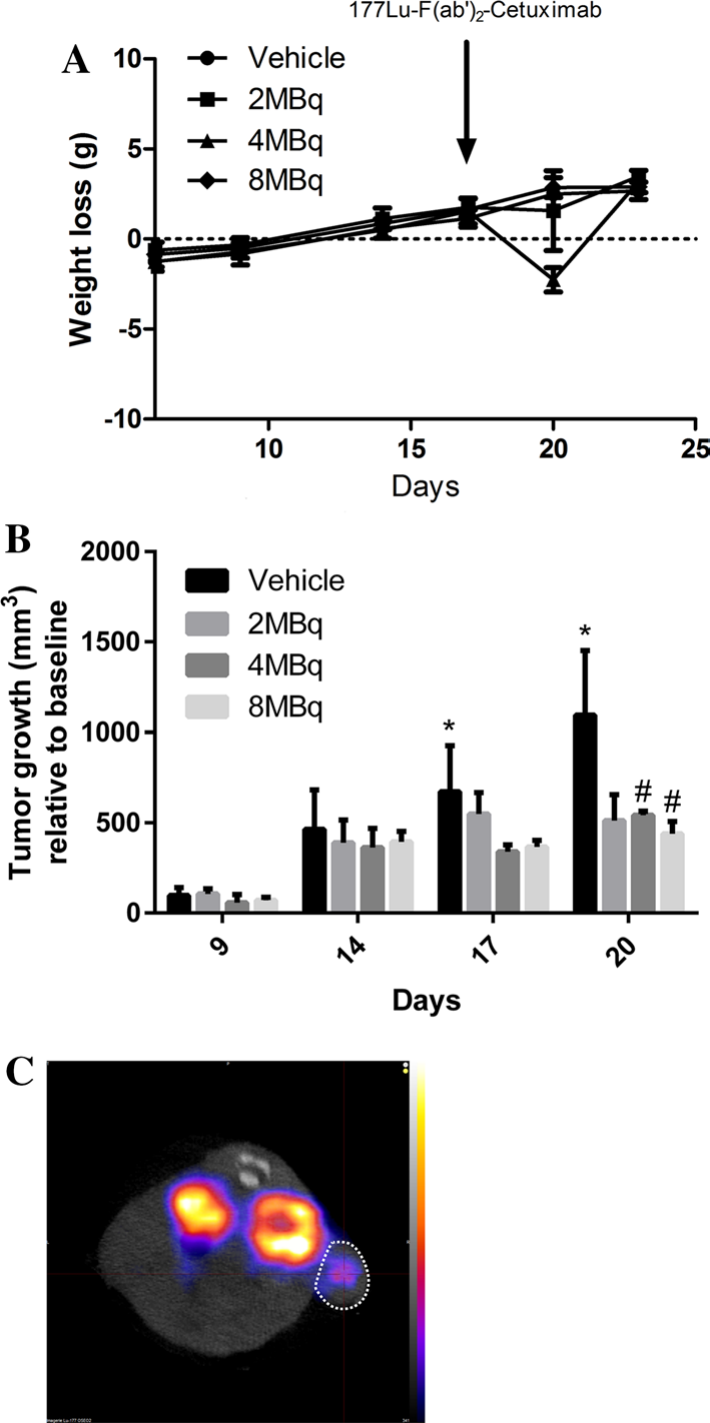
^177^Lu-DOTAGA-F(ab′)_2_-cetuximab prevents tumor growth in vivo. **a** Weight loss of Balb/c nude mice receiving vehicle or ^177^Lu-DOTAGA-F(ab′)_2_-cetuximab (2, 4 or 8 MBq). A431 cells were subcutaneously implanted at D0 and vehicle or ^177^Lu-DOTAGA-F(ab′)_2_-cetuximab injections were performed at D14. Results are presented as mean ± SEM; *n* = 4, **p < 0.01. **b** Tumor volume of Balb/c nude mice receiving vehicle or ^177^Lu-DOTAGA-F(ab′)_2_-cetuximab (2, 4 or 8 MBq). A431 cells were subcutaneously implanted at D0 and vehicle or ^177^Lu-DOTAGA-F(ab′)_2_-cetuximab injections were performed at D14. Tumor growth is expressed as a relative gain of volume from the first day when tumor was measurable (D6). Results are presented as mean ± SEM; *n* = 4, **p < 0.01. **c** Representative image of ^177^Lu-DOTAGA-F(ab′)_2_-cetuximab (4 MBq) tumor activity in a tumor-bearing nude mice (D15). White circles = tumors

SWISS nude mice grafted subcutaneously with A431 cells received a single injection of ^177^Lu- DOTAGA-F(ab′)_2_-cetuximab (iv, 2, 4 or 8 MBq) on D14 after tumor inoculation. Control mice received vehicle on D14 (iv). While both 2 MBq and 8 MBq of ^177^Lu-DOTAGA-F(ab′)_2_-cetuximab did not induce weight loss in treated animals, 4 MBq induced a rapid weight loss at D20 which was normalized by D23 (Fig. 4a). Interestingly, all three doses of ^177^Lu-DOTAGA-F(ab′)_2_-cetuximab induced a reduction of tumor growth between D14 and D20 although only significant in mice receiving 4 and 8 MBq of ^177^Lu-DOTAGA-F(ab′)_2_-cetuximab (Fig. 4b, c). Taken together these results demonstrate that ^177^Lu-DOTAGA-F(ab′)_2_-cetuximab is able to prevent A431 cells’ tumor growth with a highest efficacy at 4 and 8 MBq.

## Discussion

Colorectal cancer is nowadays the third most commonly diagnosed and the fourth leading cause of cancer-related deaths worldwide [29–31]. Current treatment options offer a limited benefit on survival of patients with stage IV colorectal cancer with a 5 years survival of less than 10%, highlighting the urgent need for innovative therapeutic strategies [31]. Theranostic approaches based on radiolabeled antibodies allow the selective targeting of tumor cell antigens for diagnostic, evaluation of therapy efficacy and delivery of ionizing radiation to tumor sites depending on the chosen radionuclide. The upregulation of EGFR has been reported in colorectal cancer and is associated with poor prognosis and resistance to radiation therapy. Thus, EGFR is nowadays an important target for therapy with anti-EGFR antibodies [32]. Cetuximab is a monoclonal antibody approved for treatment of colorectal cancer which selectively binds EGFR to prevent the binding of natural EGFR-ligands and promote antibody-receptor complex internalisation [33]. However, the pharmacokinetics of whole cetuximab is slow, due to its size, and ranges from 63 to 230 h in patients [34]. This slow pharmacokinetics is not optimal for rapid and dynamic EGFR imaging. Moreover, the extended presence of whole cetuximab in the blood often induces misinterpretation of the amount of EGFR. In mice the biological half-life of F(ab′)_2_ fragments of cetuximab is six times shorter than that of whole cetuximab (12 and 70 h, respectively [35, 36]). Thus, we designed for the current study F(ab′)_2_-cetuximab radiolabeled with ^111^Indium (^111^In-F(ab′)_2_-cetuximab) which overcomes issues raised with whole cetuximab by displaying fast blood clearance, rapid tumor accumulation and enables earlier molecular imaging [11, 37]. Our results are in accordance with previous studies which report a peak in tumor uptake of F(ab′)_2_-cetuximab at 24 h post injection [36, 37]. Moreover, the immunoreactivity and affinity of ^111^In-DOTAGA-F(ab′)_2_-cetuximab and ^111^In-DOTAGA-cetuximab found in the current study are also in accordance with the literature. Indeed, Van Dijk et al. [37] reported an immunoreactivity of F(ab′)_2_-cetuximab of 58% compared to 50% for ^111^In-DOTAGA-F(ab′)_2_-cetuximab and ^111^In-DOTAGA-cetuximab in our study. These results highlight the fact that immunoreactivity is not disturbed by DOTAGA incorporation and radiolabeling. In addition, the affinity of native cetuximab for HER1 has been reported to range from 0.62 to 1.7 nM [38, 39]. In our study, ^111^In-DOTAGA-F(ab′)_2_-cetuximab and ^111^In-DOTAGA-cetuximab displayed an affinity of 0.9 and 1.7 nM, respectively, thus demonstrating that F(ab′)_2_ fragments of cetuximab retain their affinity for HER1 after DOTAGA incorporation and radiolabeling. Therefore, F(ab′)_2_-cetuximab represents a tool of interest for theranostic application by retaining high affinity for HER1 and better pharmacokinetic properties than whole cetuximab for imaging/quantification of EGFR.

Another crucial issue in designing a radioimmunotherapeutic tool for theranostic use is the stability of the radionuclide in vitro and in vivo. Hence, the choice of the bifunctional chelating agent (BFC) used for radiolabeling is essential to minimize toxicity of the radionuclide to normal tissues. DTPA derivatives remain the most commonly used chelators for cetuximab radiolabeling in preclinical studies [9, 10] and in human [40, 41]. However, macrocyclic chelators such as DOTA derivatives have been shown to form more stable antibody-conjugates in vitro and in vivo for a wide range of radionuclides including ^111^In [20], ^177^Lu [14] and ^90^Y [18, 19]. Importantly, Camera et al. [19] reported a lower bone uptake of ^90^Y-DOTA conjugates over ^90^Y-DTPA suggesting a better in vivo stability of DOTA conjugates limiting ^90^Y toxicity. Similarly, the in vitro stability of ^177^Lu-DOTA conjugates over ^177^Lu-DTPA has been clearly demonstrated. Whether DOTA is also superior in vivo for ^177^Lu, it remains controversial. Milenic et al. [21] observed no significant differences between DOTA and DTPA conjugates, suggesting similar in vivo stabilities. On the contrary, Brouwers et al. [14] showed that uptake of ^177^Lu-DTPA was slightly higher in most tissues, including bone, and markedly higher in liver and spleen compared with ^177^Lu-DOTA suggesting a difference in in vivo stability between these two conjugates. In addition, a less complicated labeling procedure and an improved labeling efficiency often leads to a preference for DTPA as the chelator for radiolabeling with ^177^Lu for radioimmunotherapy applications [21]. Indeed, the use of DOTA derivatives induces slower complex formation rates which can limit radiolabeling yields and efficiency. In addition, radiolabeling conditions to perform complexation often require extensive timeframe and high temperatures which are not acceptable for protein conjugates [42]. Nevertheless, our group developed and characterized in a previous study a DOTAGA-anhydride chelator as a powerful tool for bioconjugation [23]. DOTAGA is a DOTA derivative that leaves four acetate pendant arms intact and can be easily synthesized in good yield. Moreover, in the current study effective conjugation of DOTAGA-anhydride to cetuximab and F(ab′)_2_-cetuximab was achieved in conditions suitable for protein conjugates. In addition, DOTAGA conjugation did not disrupt cetuximab and F(ab′)_2_-cetuximab immunoreactivity/affinity and showed greater stability than corresponding DTPA conjugates as previously described for trastuzumab [23]. Moreover, ^177^Lu-DOTAGA complex has recently been shown to be highly stable in preclinical model of prostate cancer [43] and in patients showing high efficacy and low toxicity [44, 45]. BM toxicity, including long-term haematological toxicities, has been reported in 11% of patients with metastatic neuroendocrine tumours treated with ^177^Lu-DOTA-Tyr3-octreotate (^177^Lu-DOTATATE, [46]). In addition, 40% of patients with prostate cancer receiving ^177^Lu-DKFZ-617 showed hematotoxicity in a small cohort of 10 patients [47]. Interestingly, Baum et al. [44] reported no hematotoxicity of ^177^Lu-DOTAGA-PSMA complex in patients with prostate cancer with no worsening of anemia and leukocytopenia after therapy and no grade three or four hematologic toxicity in any of the patients. Thus, DOTAGA-anhydride chelator appears as a suitable tool for bioconjugation enabling a strong sequestration of radionuclides preventing their toxicity in vivo.

In the current study, we demonstrate that ^111^In-F(ab′)_2_-cetuximab specifically target colorectal tumors expressing HER1. Our results are comparable to what was found in previous publication mainly on head and neck cancer [11, 36, 37]. Surprisingly, the tumor targeting of ^111^In-F(ab′)_2_-cetuximab in colorectal tumors remains not well documented. Van Dijk et al. [11, 37] demonstrated in a murine model of head and neck squamous cell carcinoma that ^111^In-F(ab′)_2_-cetuximab showed good tumor-to-background contrast on microSPECT imaging, allowing noninvasive assessment of EGFR expression in vivo. The same group also established that ^111^In-F(ab′)_2_-cetuximab was able to monitor the effects of EGFR inhibition or irradiation much better than ^18^F-FDG PET confirming the added value of ^111^In-F(ab′)_2_-cetuximab to follow treatment efficacy [48]. In our study, ^111^In-DOTAGA-F(ab′)_2_-cetuximab was used as a diagnostic tool for colorectal cancer to follow the efficacy of a HER1 targeted therapy by the HSP90 inhibitor, 17-DMAG. HSP90 is a chaperon protein that has been demonstrated to bind and stabilize HER1 and whose inhibition causes a decrease in EGFR in cancer cells [27]. In addition to reducing membrane expression of EGFR, HSP90 inhibition significantly prevented tumor growth in head and neck squamous cell carcinoma animal models [27]. Interestingly, Spiegelberg et al. [49] recently showed by PET imaging with ^124^I-labeled cetuximab that HSP90 inhibition induced a decreased in EGFR expression in head and neck squamous cell carcinoma. Our results confirm, in a colorectal cancer model, that SPECT imaging of ^111^In-F(ab′)_2_-cetuximab is able to monitor EGFR downregulation and the prevention of tumor growth caused by HSP90 inhibition in vivo. Indeed, HSP90 inhibition induced a decrease in tumor uptake as well as a decrease in tumor growth compared to control mice. Interestingly, we demonstrate here that tumor volume physically measured with a clipper strongly correlated with tumor volumes assessed directly by SPECT imaging of ^111^In-F(ab′)_2_-cetuximab further validating the accuracy of imaging to follow tumor growth upon targeted therapy. Thus, tumor uptakes can be accurately expressed in regards to tumor volumes assessed directly by SPECT imaging in % ID/mm^3^. However, a statistical difference between 17-DMAG and control group was found from D44 when tumor volumes were manually measured, while significance was reached only from D51 when tumor volumes were measured by SPECT imaging. Due to its specificity, imaging should be more accurate than manual measurements but in our experiments only four mice per group were imaged while ten mice per group were used for manual measurements. This difference in the number of animal can explain the discrepancy between SPECT imaging and manual measurements in the time to reach significance.

One of the advantages of the ^111^In-F(ab′)_2_-cetuximab probe is the possibility to perform a radionuclide switch from ^111^In to ^177^Lu to obtain a therapeutic tool ^177^Lu-F(ab′)_2_-cetuximab. β^ −^  particles emitted by ^177^Lu have a medium energy and a subsequent tissue penetration of 1.5 mm supporting its therapeutic use favored in treatment of small tumors while limiting irradiation of normal tissue [50]. Another asset of ^177^Lu, in comparison to other therapeutic radionuclide such as ^90^Y, is the low energy γ-emission that allows imaging. The combination of imaging and therapeutic properties makes ^177^Lu a theranostic tool of choice increasingly used in preclinical and clinical studies [15, 44, 51, 52]. We demonstrate here that radioimmunotherapy with ^177^Lu-F(ab′)_2_-cetuximab significantly inhibited colorectal tumor growth when 4 and 8 MBq are injected, but not significantly at 2 MBq. Our results confirm a previous study performed by Song et al. [16] with the whole form of cetuximab in a model of esophageal cancer. As mentioned above ^177^Lu induces myelotoxicity. Interestingly, even if we did not evaluate hematologic toxicity, we noticed weight loss in mice receiving 4 MBq 6 days after injection, suggesting toxicity. Our results are in accordance with the findings of Fischer et al. [51] who evaluated experimentally the maximal tolerated dose of ^177^Lu in nude mice bearing human ovarian cancer around 12 MBq. Nevertheless, even if 4 MBq seemed to show early signs of toxicity (with no effect on survival), other doses (2 and 8 MBq) did not induce toxicity and showed efficacy in reducing tumor growth. Thus, a dose of 2 MBq could be sufficient to achieve significant efficacy on tumor growth prevention without toxicity. The low energy and a low tissue penetration of ^177^Lu favored its use for treatment in metastatic cancer. This could be of great interest in the treatment of peritoneal carcinomatosis of colorectal cancer origin in which EGFR upregulation has been demonstrated to be a factor of poor prognosis [53]. Cetuximab is already widely used for metastasized colorectal cancer but no clinical data on its specific use in peritoneal carcinomatosis is nowadays available [53]. ^177^Lu-F(ab′)_2_-cetuximab could allow peritoneal carcinomatosis early diagnosis by localizing metastasis formation and could also represent an important therapeutic strategy by specifically delivering ionizing radiation during early metastasis invasion.

## Conclusion

To conclude, we demonstrate in the current paper that DOTAGA-F(ab′)_2_-cetuximab exhibits high in vivo and in vitro stability after radiolabeling with ^111^In and ^177^Lu. ^111^In-DOTAGA-F(ab′)_2_-cetuximab is a reliable tool for SPECT imaging of colorectal cancer overexpressing HER1 and enables accurate therapy efficacy monitoring (e.g. anti-HSP90 therapy). Moreover, the radionuclide switch from ^111^In to ^177^Lu to form ^177^Lu-DOTAGA-F(ab′)_2_-cetuximab allows radioimmunotherapy coupling therapeutic efficacy and accurate imaging properties to treat and image HER1 expressing colorectal tumors.

## Electronic supplementary material

Below is the link to the electronic supplementary material.

Supplementary material 1 (DOCX 24 kb)

## Abbreviation

Fab: Antigen-binding fragment
SPECT: Single photon emission computed tomography
DOTAGA: 2,2′,2′′-(10-(2,6-Dioxotetrahydro-2H-pyran-3-yl)-1,4,7,10-tetraazacyclododecane-1,4,7-triyl)triacetic acid
CT: Computed tomography
SEM: Standard error of mean
EGFR: Epidermal growth factor receptor
17-DMAG: 17-(Dimethylaminoethylamino)-17-demethoxygeldanamycin
^111^In: Indium-111
^177^Lu: Lutetium-177
HSP: Heat shock protein
HNSCC: Head and neck squamous cell carcinoma
DOTA: 1,4,7,10-Tetraazacyclododecane-1,4,7,10-tetraacetic acid
DTPA: Diethylenetriamine-penta-acetic acid
BFCA: Bifunctional chelating agent
