# Supplementary material for: Radiolabeled F(ab′)2-cetuximab for theranostic purposes in colorectal and skin tumor-bearing mice models
Source: Clin Transl Oncol. 2018 May 17;20(12):1557–70. doi: 10.1007/s12094-018-1886-4 (PMC6223717; doi:10.1007/s12094-018-1886-4)
Supplement: Supplementary file 1 — Supplementary material 1 (DOCX 24 kb) [file 12094_2018_1886_MOESM1_ESM.docx]

**Supplemental Material and methods**

***Preparation of cetuximab and F(ab’)2 fragments***

Cetuximab was prepared by concentration of 30 mL of cetuximab at 5 mg.mL^-1^ on 10 kDa cut off (Amicon Ultra 15 – Millipore) using centrifugation (4000 g; 3 x 20 min) and dialysis 48h against PBS (137 mM NaCl, 2.7 mM KCl, 1.5 mM KH_2_PO_4_, 12 mM Na_2_HPO_4_ pH 7.4).

F(ab)’_2_ fragment was prepared by dialysis of 15 mL of cetuximab at 5 mg.mL^-1^ against pepsin buffer (sodium acetate 20 mM/ acetic acid pH4) on 10 kDa cut off (Amicon Ultra 15 – Millipore) using centrifugation (4000 g ; 2 x 20 min). Dialysis products were diluted at a final concentration of 4 mg.mL^-1^ with pepsin buffer. Then, cetuximab was digested by pepsin with 1/50 ratio (enzyme/antibody concentration) during 8h at 37°C in a water bath. Reaction was stopped with 1/10 (v/v) Tris 2M pH11.2 and digestion products were dialyzed 48h against PBS. Sample were purified on protein A column (GE Healthcare) in order to eliminate whole antibody that was not digested by pepsin. Then, unbound and first wash fractions were passed through protein L column (GE Healthcare) in order to purify F(ab)’_2_ fragment which was finally dialyzed 48h against PBS.

Cetuximab and F(ab)’_2_ fragments concentration was determined at 280 nm and purity was analyzed on 4-12% bis-tris acrylamide gel (Nupage - Invitrogen) with Coomassie blue staining (sample: 5 µg).

***Derivatization of cetuximab and F(ab’)_2_ fragments for radiolabeling***

*Conjugation of DOTAGA anhydride to cetuximab.*

Conjugation was performed at a 20:1 DOTAGA-anhydride/cetuximab molar ratio. 100 μL of a 3.7 mg.mL^−1^ suspension of DOTAGA-anhydride (370 μg, 0.8 μmol, 20 equiv) in dry chloroform (Carlo Erba, Val de Reuil, France) were pipetted under ultrasonication and transferred into a 15 mL polypropylene tube. The chloroform was evaporated under a gentle stream of air. 480 μL of a solution of purified cetuximab (12.5 mg.mL^−1^, 6 mg, 40 nmol, 1 equiv) in PBS, pH 7.4, (Fisher Scientific, Illkirch, France) was subsequently added. The solution was completed to 3 mL with PBS 0.1 M, pH 7.4, and gently mixed at 25°C for 30 min. Unbound DOTAGA was then removed by ultrafiltration (Vivaspin filter 10 kDa, Sartorius, 30 min at 1520 *g*, 4°C). Conjugated cetuximab was washed twice with 5 mL of PBS 0.1 M, pH 7.4, and the concentrated solution was diluted in 500 μL of ammonium acetate buffer 0.1 M pH 5.9. The purified immunoconjugate DOTAGA-cetuximab was stored at 4°C. Concentration of the antibody was determined by UV spectrophotometry at 280 nm (E_280_ = 1.44 mg.mL^-1^.cm^-1^). Degree of labeling was determined by MALDI-TOF mass spectrometry using sinapinic acid as matrix.

*Conjugation of DOTAGA anhydride to F(ab’)_2_-cetuximab.*

Conjugation was performed at a 15:1 DOTAGA-anhydride/F(ab’)_2_ molar ratio. 111 μL of a 3.7 mg.mL^−1^ suspension of DOTAGA-anhydride (411 μg, 0.9 μmol, 15 equiv) in dry chloroform were pipetted under ultrasonication and transferred into a 15 mL polypropylene tube. The chloroform was evaporated under a gentle stream of air. 1.39 mL of F(ab’)_2_-cetuximab (4.3 mg.mL^−1^, 6 mg, 60 nmol, 1 equiv) in PBS, pH 7.4, was subsequently added. The solution was completed to 3 mL with PBS 0.1 M, pH 7.4, and gently mixed at 25°C for 30 min. Unbound DOTAGA was then removed by ultrafiltration (Vivaspin filter 10 kDa, Sartorius, 30 min at 1520 *g*, 4°C). Conjugated F(ab’)_2_-cetuximab was washed twice with 5 mL of PBS 0.1 M, pH 7.4, and the concentrated solution was diluted in 500 μL of ammonium acetate buffer 0.1 M pH 5.9. The purified immunoconjugate DOTAGA-F(ab’)_2_-cetuximab was stored at 4°C. Concentration of the immunoconjugate was determined by UV spectrophotometry at 280 nm (E_280_ = 1.44 mg.mL^-1^.cm^-1^). Degree of labeling was determined by MALDI-TOF mass spectrometry using sinapinic acid as matrix.

***^111^In and ^177^Lu radiolabeling procedures***

*General procedure for radiolabeling for in vitro studies.*

7.5 MBq of ^111^InCl_3_ (Perkin Elmer) were added to 50 μg of the immunoconjugate in 0.1 M ammonium acetate buffer, pH 5.9, to reach a buffer/HCl (from ^111^InCl_3_ solution) ratio of 1.5:1 resulting in a pH 5 solutions. The solution was stirred in a thermomixer at 37 °C during 1 h. After incubation, 50 mM EDTA in 0.1 M ammonium acetate was added in order to chelate free ^111^In. The resulting ^111^In-EDTA was then removed by ultrafiltration, and the product was diluted in PBS 0.1 M, pH 7.4. Instant thin layer chromatographies (ITLC) were performed before and after ultrafiltration to determine the radiolabeling yield and to assess the absence of free ^111^In. 1 μL of each solution was deposited on ITLC-SG strips at 2 cm of the bottom. The eluent (sodium citrate 0.1 M, pH 5) was allowed to rise to 10 cm from the bottom of the strips. Radiolabeled antibody remained at the application point while free ^111^In or ^111^In-EDTA migrated with solvent front. The strips were then analyzed using a γ radiochromatograph.

*General procedure for radiolabeling for in vivo studies.*

^111^InCl_3_ or ^177^LuCl_3_ (Perkin Elmer) were buffered with 1/10^th^ (v/v) of 1 M ammonium acetate solution pH 7.1 and then added to DOTAGA-F(ab’)_2_ in 0.1 M ammonium acetate buffer pH 5.9 (600 MBq.mg^-1^ and 1 GBq.mg^-1^ respectively). The solution was stirred for 2h at 37°C. After incubation, 50 mM EDTA in 0.1 M ammonium acetate was added (1/10^th^ (v/v)) in order to chelate free radionuclide. After purification by ultrafiltration, the purity was verified by ITLC as previously described, and the radiolabeled antibody was diluted in PBS 0.1 M, pH 7.4, for injection.

***Stability assay***

^111^In-DOTAGA-F(ab’)_2_-cetuximab/^111^In-DTPA-F(ab’)_2_-cetuximab stability in EDTA and plasma were evaluated up to 7 days. Briefly, 10 µL aliquots of each compound were mixed with 90 µL of EDTA (1000 eq)or human plasma and incubated at 37°C. At each time point, the percentage of ^111^In activity dissociated from DOTAGA-F(ab’)_2_-cetuximab and DTPA-F(ab’)_2_-cetuximab was assessed by ITLC-SA using EDTA 0.1 M, pH 5 as the mobile phase. Serum aliquots (1-2 µL) were spotted on the chromatography paper and allowed to run 10 cm from the origin. Radiolabeled antibody remained at the application point while transchelated ^111^In migrated with solvent front. The strips were then analyzed using a γ radiochromatograph.

***Cell culture***

Epidermoid carcinoma cells A431 (ATCC, Rockville, MD) overexpressing the HER1 antigen at their surface ([*28*](#_ENREF_28)) and primary human cells from colon tumor (CR-LRB-014P) have been used. CR-LRB-014P cells have been collected from a biopsy of a primitive colorectal tumor from a 70 years old male in 2008 in the hospital center of “Lariboisière”, Paris, France (no treatment before surgery). Cells were grown as adherent monolayer in RPMI1640 medium (Lonza, Belgium) supplemented with 10% fetal calf serum (Lonza). Cells were harvested just prior to experiments. For *in vitro* experiments, cells were washed twice with PBS 0.1 M pH 7.4 (Lonza) and resuspended in PBS 0.1 M pH 7.4 containing 0.2% bovine serum albumin (BSA) and 0.02% azide (Sigma, France). For *in vivo* experiments, cells were washed twice with RPMI1640 medium and resuspended in a mixture of RPMI1640 and matrigel (BD Biosciences) (1/1, v/v). Before all experiments, the viability of the cells was assessed by trypan blue exclusion assay. In all cases, viability was greater than 90%.

***Determination of ^111^In-DOTAGA-cetuximab and ^111^In-*DOTAGA-F(ab’)_2_-cetuximab** ***binding affinity and immunoreactivity***

*Immunoreactivity*

The fraction of ^111^In-DOTAGA-F(ab’)_2_-cetuximab and ^111^In-DOTAGA-cetuximab able to bind to HER1 was determined by incubating trace amounts of ^111^In-DOTAGA-F(ab’)_2_-cetuximab or ^111^In-DOTAGA-cetuximab (1MBq, 7.0 × 10^−9^ M) with increasing concentrations of HER1 expressing cell A431 (0.4 to 24 × 10^6^ cells.mL^−1^) in a total volume of 0.2 mL for 1h at 4°C. At the end of the incubation period, cells were centrifuged, rinsed twice with ice-cold PBS (0.1 M pH 7.4), and then dissolved in 0.1 N NaOH. The radioactivity associated to cells (bound radioactivity, B) and an aliquot of the supernatant (total radioactivity, T) were counted with a scintillation γ counter (Cobra 4180, Perkin-Elmer Inc.) in order to calculate the bound-to-total ratios (B/T, expressed in %). Nonspecific binding was evaluated in the presence of a >100-fold excess unlabeled cetuximab. Immunoreactivity was defined as the highest B/T% ratio that could be reached.

*Binding affinity*

The affinity constant (Kd) of the ^111^In-DOTAGA-F(ab’)_2_-cetuximab and ^111^In-DOTAGA-cetuximab were determined in radioligand binding saturation assays. Approximately 3 × 10^5^ A431 cells were incubated with increasing concentrations of ^111^In-DOTAGA-F(ab’)_2_-cetuximab or ^111^In-DOTAGA-cetuximab (5.3 × 10^−11^ to 5.4 × 10^−8^ M, 100 MBq.mg^−1^) in a total volume of 0.2 mL for 1h at 4°C. At the end of the incubation period, cells were centrifuged, rinsed twice with ice-cold PBS 0.1 M pH 7.4, and then dissolved in 0.1 N NaOH. Radioactivity associated to cells was counted with a scintillation γ-counter. Nonspecific binding was evaluated in the presence of a >100-fold excess unlabeled DOTAGA-F(ab’)_2_-cetuximab or DOTAGA-cetuximab. The dissociation constant was determined from experimental results by curve-fitting using GraphPad Prism v 5.04 for Windows (GraphPad Software, San Diego, California, USA).

***Animal models***

All animal experiments were performed according to the guidelines of the Ministère de la Recherche (Paris, France). All experiments were approved by the ethical committee of the “Centre George François Leclerc” (Dijon, France).

*Biodistribution study: SPECT/CT imaging protocol and γ-counting*

Female Balb/c nu/nu mice (6−8 weeks old, purchased from Charles River, France) were grafted by subcutaneous injection of colon tumor fragments from human patients (CR-LRB-014P). When grown these tumors were collected and fragments from these tumors were implanted into a second set of Balb/c nu/nu mice (6−8 weeks old). Three or five weeks after tumor implantation, tumor bearing-mice were given 25 μg ^111^In-DOTAGA-F(ab’)_2_-cetuximab (13−15MBq) by intravenous injection. In a second experiment to assess the specificity of the targeting *in vivo*, a group of mice received 25 μg ^111^In-DOTAGA-F(ab’)_2_-cetuximab (3−3.5 MBq) in co-injection with excess (2500 μg) cold- F(ab’)_2_-cetuximab. SPECT/CT dual imaging was performed 3, 6, 20, 24, 48, and 72h after the injection of the radiolabeled conjugate using a NanoSPECT/CT small animal imaging tomographic γ-camera (Bioscan Inc., Washington, DC). Mice were anaesthetized with isoflurane (1.5−3% in air) and positioned in a dedicated cradle. CT and SPECT acquisitions were performed in immediate sequence. CT acquisitions (55 kVp, 34 mAs) were first acquired during 15−20 min, followed by helical SPECT acquisitions with 90−120s per projection frame resulting in acquisition times of 45−60 min. Both indium-111 photopeaks (171 and 245 keV) were used with 10% wide energy windows. After the last image acquisition, animals were euthanized. Blood, tumor, and organs were collected, and radioactivity was measured with a scintillation γ-counter. Data were then converted to percentage of injected dose and to percentage of injected dose per mm^3^ of tissue (the injected doses being corrected for subcutaneously injected material remaining in the animal tail). The CT and SPECT reconstructions were performed using image processing software provided by Bioscan Inc. Eventually, the SPECT/CT fusion image was obtained using the InVivoScope software (Bioscan Inc.). Each scan was then visually interpreted, and 3D regions of interest corresponding to the tumor and whole body were manually drawn in order to determine their radioactivity content. *In vivo* quantification was obtained by accurate calibration of the NanoSPECT/CT γ-camera. Radioactivity contents from image analysis were expressed in Bq/mm^3^, converted to percentage of injected dose, and compared to those determined by *ex vivo* counting.

*^111^In-DOTAGA-F(ab’)_2_-cetuximab for therapy evaluation*

Female Balb/c nu/nu mice (6−8 weeks old) were grafted by subcutaneous injection of colon tumor fragments from human patients. When grown these tumors were collected and fragments from these tumors were implanted into a second set of Balb/c nu/nu mice (6−8 weeks old). Tumor volume was measured 3 times a week from D30 to D50 after cells injection. Mice were randomized and received i.p. 17-DMAG (25 mg/kg) or vehicle 3 times a week from D30. ^111^In-F(ab’)2-cetuximab tumor uptake (i.v. injection) was evaluated every week from D30 using SPECT-CT imaging (20-25 μg, 10 MBq, imaging 24h after injection) as described above (D38, D44, D51 and D58).

*Dose escalation of ^177^Lu-DOTAGA-F(ab’)_2_-cetuximab in vivo*

Under isoflurane anesthesia, female SWISS nu/nu mice were grafted by subcutaneous injection in the flank with 2 × 10^7^ A431 cells. Tumor volume was measured 3 times a week from D3 after administration. At D14 after tumor cell injection, mice were randomized and ^177^Lu-DOTAGA-F(ab’)_2_-cetuximab was injected i.v. (vehicle, 2MBq, 4MBq or 8MBq/mouse). SPECT-CT imaging was performed 24h after i.v. injection as described above to assess tumor targeting. Tumor volume was then assessed 3 times a week up to D23. Weight loss of animals was monitored throughout the experiments.

***Statistical analysis***

All results are presented as mean ± SEM. Statistical analysis between two groups was performed using a parametric Student’s t test. Statistical analysis between multiple groups with one control group was performed by a one way ANOVA test, with Tukey’s multiple comparison test (post hoc). Analysis were performed with GraphPad Prism 6.0 (GraphPad Software Inc.), in all cases a p-value less than 0.05 was considered significant.
